# Tangram of Sodium and Fluid Balance

**DOI:** 10.1161/HYPERTENSIONAHA.123.19569

**Published:** 2023-12-12

**Authors:** Domenico Bagordo, Gian Paolo Rossi, Christian Delles, Helge Wiig, Giacomo Rossitto

**Affiliations:** Emergency and Hypertension Unit, Dipartimento di Medicina (DIMED), Università degli Studi di Padova, Italy (D.B., G.P.R., G.R.).; School of Cardiovascular & Metabolic Health, University of Glasgow, United Kingdom (G.R., C.D.).; Department of Biomedicine, University of Bergen, Norway (H.W.).

**Keywords:** body fluids, electrolytes, homeostasis, hypertension, water

## Abstract

Homeostasis of fluid and electrolytes is a tightly controlled physiological process. Failure of this process is a hallmark of hypertension, chronic kidney disease, heart failure, and other acute and chronic diseases. While the kidney remains the major player in the control of whole-body fluid and electrolyte homeostasis, recent discoveries point toward more peripheral mechanisms leading to sodium storage in tissues, such as skin and muscle, and a link between this sodium and a range of diseases, including the conditions above. In this review, we describe multiple facets of sodium and fluid balance from traditional concepts to novel discoveries. We examine the differences between acute disruption of sodium balance and the longer term adaptation in chronic disease, highlighting areas that cannot be explained by a kidney-centric model alone. The theoretical and methodological challenges of more recently proposed models are discussed. We acknowledge the different roles of extracellular and intracellular spaces and propose an integrated model that maintains fluid and electrolyte homeostasis and can be distilled into a few elemental players: the microvasculature, the interstitium, and tissue cells. Understanding their interplay will guide a more precise treatment of conditions characterized by sodium excess, for which primary aldosteronism is presented as a prototype.

Historically, studies and public policies on cardiovascular disease and particularly hypertension have focused on sodium (Na^+^) intake, while relatively less attention has been devoted to differences in its handling. Old assumptions on the constancy of the internal environment have now been challenged, revealing that Na^+^ balance can be dynamic and over time not even perfectly balanced.

Approximately 10 years ago, Bhave and Neilson^1^ reviewed the mechanisms of body fluid and electrolyte dynamics in relation to body compartments and reconnected historical principles with novel insights. In this review, we will expand on those dynamics, presenting new technologies and evidence, but also old experimental data that provide ground to our current interpretations. In particular, we will discuss the following: (1) the variability of day-to-day Na^+^ balance; (2) the concept of long-term uneven Na^+^ balance in cardiovascular and renal disease, long established but nowadays directly visualized as Na^+^ excess in tissues; (3) the evolving interpretation for such tissue signal; (4) the importance of interstitial fluid balance, microvascular interface, and intracellular compartment. In these regard, insights from primary aldosteronism (PA), a prototypic salt-sensitive disease leading to hypertension but also to cardiorenovascular damage in excess of blood pressure (BP) values, will be presented.

## ROLLERCOASTER BALANCE

Urinary Na^+^ excretion has traditionally been assumed to invariably reflect intake. However, the day-to-day validity of such equivalence has been recently disproved by long-term balance studies^2,3^: the daily Na^+^ excretion showed remarkable oscillations around the amount of salt targeted by the dietitians (Figures S1 and S2), in addition to weekly and monthly periodicity related to rhythmic hormonal control.^3^ The daily Na^+^ excretion from 1 single 24-hour urine collection predicted the recorded Na^+^ intake within a predefined±25 mmol 2-sided interval only in 49% of cases. Reassuringly, the average daily Na^+^ excretion provided an accurate estimate of mean salt intake, thus reflecting the ultimate achievement of a steady-state balance, and repeated 24-hour collections (but not nocturnal only^4^ or spot urine^5^) improved the precision.^3,6^ In keeping with a variability of 24-hour collections, half of the subjects included in a single-center cohort study switched between tertiles of estimated Na^+^ intake at up to 15-year follow-up collections, compared with a reference baseline. With the limit of a noninterventional design and lack of control on dietary changes, disease, and medications over time, this reclassification significantly affected the observed relationship between Na^+^ intake and long-term cardiovascular and renal outcomes.^7^ Averaging multiple collections strengthened the association,^7^ as later confirmed.^8^

Should we abandon the use of 24-hour urine collections for the estimation of daily Na^+^ intake in patients? We should not, because of 2-fold considerations. First, single 24-hour urine collections from adequately sized cohorts offer a precise estimate of group—albeit not of individual—Na^+^ intake,^9^ which is relevant for researchers and study designs. Second, if the goal of sensible clinicians is to detect true excess intake and not a precise value, to avoid false positives and consequent unnecessary and potentially stressful changes in dietary habits, the focus should be on a 1-sided end, rather than 2-sided CI. This clinical approach requires less precision: a 270 mmol Na^+^/day, for example, is unlikely to reflect anything but high Na^+^ intake, which is universally recognized as a cardiovascular risk factor.^10^ In the setting of our hypertension clinics where dietary advice and medications are controlled,^11^ 24-hour urine collection remains a valuable and inexpensive tool to decipher the biochemical screening of secondary forms,^12^ to estimate adherence to lifestyle recommendations and to provide semiquantitative metrics for positive patient reinforcement upon clinically significant reductions at follow-up, provided that data are interpreted with the patient and cum grano salis—much welcome in this occasion.

## TANGRAM

### Ultra-Long-Term or Life-Long Balance May Obey Different Rules

**Figure 1.**
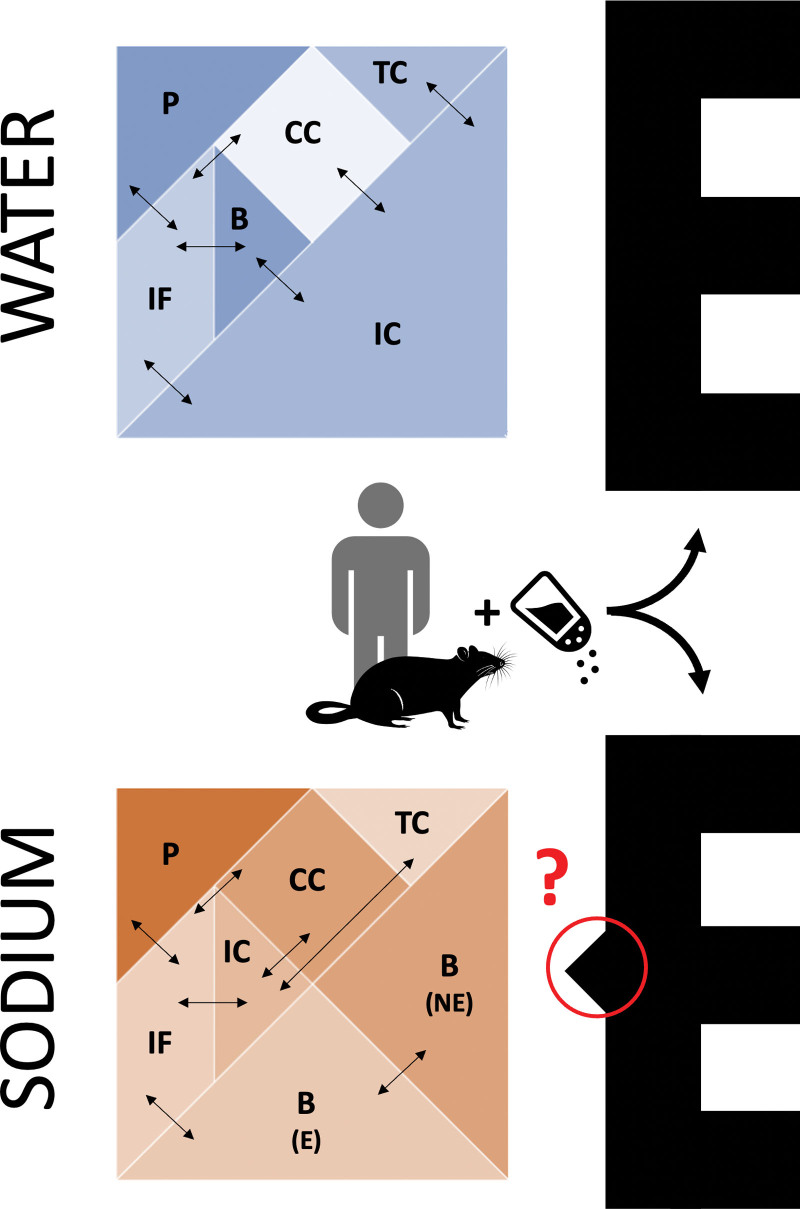
**The Tangram of water and sodium.** The Tangram shapes (**left**) depict the traditional distribution of body water and sodium in compartments.^20^ Experimental salt loading of humans and rats led to a puzzling excess of sodium compared with water,^13–17^ which reminds of Tangram paradoxes (E shapes): 2 figures composed with the same 7 pieces, one of which incomprehensibly seems to be a subset of the other (solution shown in Figure S3). B indicates bone; CC, dense connective tissue and cartilage; E, exchangeable fraction; IC, intracellular; IF, interstitial/lymphatic fluid; NE, nonexchangeable fraction; P, plasma; and TC, transcellular.

Long-term human balance studies, in keeping with previous experimental reports of positive Na^+^ (but not water) balance on extremely high Na^+^ intake,^13^ found considerable changes in total body Na^+^ without parallel changes in body weight.^2,14^ Similarly, multiple studies in rodents identified a dissociation between Na^+^ and water tissue content.^15–17^ The degree of Na^+^-associated fluid retention upon high Na^+^ intake was first shown to differ in animal models of normotension, salt-resistant, and salt-sensitive hypertension, with the former showing the highest water-independent Na^+^ storage capacity.^15^ Subsequent body composition studies, conducted by desiccation and ashing of whole rat carcasses or specific parts (ie, bones, quadriceps muscles, and skin), suggested the skin as the main depot for excess Na^+^ accumulation.^16–19^ If one sees the analogy of body fluid and electrolyte compartmentalization^20^ with a Tangram, an old dissection puzzle consisting of a few pieces that are variably combined to form different shapes, these unphysiological findings of a water-free Na^+^ excess would seem like one of those Tangram paradoxes with seemingly redundant (or missing, depending on one’s perspective) pieces (Figure 1).

The most obvious answer to the question “where is the salt?”^21^ pointed to the extracellular space, that is, the compartment where 98% of total body Na^+^ is confined.^20^ A volume-independent hypertonic extracellular accumulation of Na^+^ appeared most likely, as volume-paralleled extracellular expansion was at odds with the unchanged extracellular volume measured across different Na^+^ intake phases in the original reports.^13^ Although the magnitude of the increase in Na^+^ intake between experimental groups and the inulin-based measure of extracellular volume have been criticized,^22^ the hypothesis of a hypertonic interstitium was strengthened by evidence of TonEBP (tonicity-responsive enhancer-binding protein) activation in the skin resident mononuclear phagocytic cells of salt-loaded rodents.^18^ TonEBP-mediated signaling included Vascular Endothelial Growth Factor-C (VEGF-C) secretion, VEGF receptor 3 activation, and plastic expansion of the lymphatic vascular network, to provide enhanced local Na^+^ excess clearance.^19^ Disruption of this pathway resulted not only in skin Na^+^ accumulation but also in salt-sensitive hypertension.^19,23^ These findings led to conclude that water-independent binding of Na^+^ to the negatively charged glycosaminoglycan network, particularly represented in the skin interstitium and expanded by dietary NaCl loading,^24^ could explain the puzzling tangram appendage (Figure 1), that is, where the retained sodium was being stored. The plasticity of glycosaminoglycans in regulating tissue Na^+^ binding and storage was supported by subsequent rat^25,26^ and human^27^ data, including distinct responses in animals and patients with genetically altered glycosaminoglycan structure.^28,29^ The concept of glycosaminoglycan binding and osmotic inactivation of Na^+^ is, however, problematic because this would rely on repulsion and thereby excretion of chloride (Cl^−^),^30^ found to be increased rather than reduced in rat skin during high-salt conditions.^19^ Moreover, experiments designed to assess glycosaminoglycan binding of Na^+^ in skin indicated that such binding was negligible.^31^ Recent observations in rats and mice also showed no increase in skin glycosaminoglycans by salt loading, or even a decrease with mineralocorticoid (deoxycorticosterone acetate [DOCA]) treatment, despite significant tissue Na^+^ storage.^31,32^ These heterogeneous observations may be explained by methodological differences or by additional players such as inflammation and mechanical stress (shown to induce glycosaminoglycan production in cardiac fibroblasts^33^ and skin^34^) rather than a solely Na^+^-dependent control of glycosaminoglycans.^35^ While the implication of interstitial mucopolysaccharides in the regulation of circulation dates back to Guyton et al,^36^ how this links to tissue Na^+^ and the direction of the reported associations still remain unclear.

## I WAS BLIND BUT NOW I SEE (JOHN 9:25)

The development of ^23^Na magnetic resonance spectroscopy and high-magnetic field imaging (MRI) in the last decade has given us the chance of seeing Na^+^.

^23^NaMRI, originally validated against direct chemical analysis of tissues and calibrated with phantoms containing NaCl at different concentrations,^37^ revealed excess skin and muscle Na^+^ content in patients with resistant hypertension or PA,^38,39^ diabetes,^40–42^ heart failure,^43,44^ but also systemic inflammatory conditions,^45–48^ lipedema,^49^ and obesity, but only in the presence of high circulating inflammatory markers.^50^ Chronic kidney disease (CKD), a highly salt-sensitive condition, has been the most extensively studied: patients on maintenance hemodialysis, particularly those with concomitant diabetes,^51^ harbor high tissue Na^+^ content,^52–54^ comparable to heart failure.^55^ Tissue Na^+^ in patients with CKD was higher than in controls even before the end stage^56^ and correlated with left ventricular mass better than total body overhydration or BP.^57^ A similar association of tissue Na^+^ with target organ remodeling, independent of age, gender, diuretic use, and 24-hour ambulatory BP, has been found in diabetes for retinal vessels.^58^ When Bhave and Neilson^1^ contended that only about 5% of essential hypertension in American patients may involve alterations in (tissue) Na^+^ storage, that is, those on a diet >300 Na^+^ mEq/day, they could not know this pandemic scale of tissue Na^+^ excess, even exceeding values found in hypertensives, as later revealed by ^23^NaMRI.^41^

Nonetheless, a few related technical aspects are worth additional considerations. Similar to most reported chemical analyses of homogenized tissues, only recently coupled with a reliable extracellular volume tracer to provide some compartmental information,^31^ current ^23^NaMRI can only measure whole-tissue Na^+^. In fact, it was recently claimed that ^23^NaMRI protocols used in the clinical setting predominantly reveal signals from free (dissolved) ions,^42,59^ thus lending scarce support to the hypothesis of excess glycosaminoglycan-bound Na^+^ in cardiovascular disease; however, whether it is possible to reliably discriminate the amount of Na^+^ that is free or bound (and this definition may extend to the intracellularly constrained pool) remains much debated.^59^ For sure, and at odds with the suggested hypertonicity, all authors reported that whole-tissue Na^+^ levels for the above pathologies are ≤40 mmol/L, far below the Na^+^ concentration typically found in plasma,^1^ but also in lymph or interstitial fluid eluate.^60^ This may reflect the presence of a relatively Na^+^-poor intracellular fraction in all tissues, which would dilute the signal from the interstitium in the final whole-tissue readout.^61,62^ Unfortunately, the attempt to separate the intracellular from extracellular fractions is limited by MRI spatial resolution and by a similar relaxation time of Na^+^ in the 2 compartments^63^ or by toxicity of extracellular-limited shifting agents.^64^ To date, multinuclear, multicompartment modeling, which has shown promise, is still in its infancy and not devoid of limitations.^59,65^ The feasibility of tissue Na^+^ imaging down to a cellular scale has recently been proven with the alternative approach of X-ray fluorescence spectromicroscopy, but the technique is destructive and yielded extracellular and intracellular concentrations opposite to what is expected from physiology, likely due to the chemical treatment used for the sample preparation.^66^ Therefore, while waiting for validated and clinically applicable advancements, the available ^23^NaMRI data should be interpreted as total tissue Na^+^.

## ON THE SHOULDERS OF GIANTS

Our predecessors were blind but knowledgeable. Not only did they deduce morphological information on tissues and their constitutive compartments from purely chemical measurements since the 40s but they also knew that tissue electrolytes change with aging, toward excess Na^+^ and Cl^−^ (and loss of K^+^).^67–70^ Moreover, in vivo studies from the 70s and 80s performed with nuclear whole-body counting, revealed high total and exchangeable Na^+^ (NaE) retained somewhere in the body of patients with many of the conditions in which ^23^NaMRI was later applied, including some forms of hypertension. Those studies, at the time conducted in patients with overt increase in BP but on no treatment or in adequate washout, showed that excess body Na^+^ (or Cl^−^) does not necessarily involve all patients with hypertension at any stage of the disease.^71,72^ In particular, NaE was significantly increased in PA but was normal in patients presumed to have essential hypertension and in those with unilateral renal artery stenosis or even below normal in young hypertensives aged ≤35 years.^73^ Nevertheless, the correlation of NaE with arterial pressure was positive and significant in almost all patients with hypertension,^71,73^ as for NaE and total body Na^+^ (*r*=0.91; *P*<0.001).^71^ Modern ^23^NaMRI studies overall confirmed these findings, suggesting that young, noncomorbid subjects with early-stage hypertension may not show any increase in tissue Na^+^, at variance with resistant hypertension or PA,^38,41,74^ although definitive conclusions are prevented by limitations including sample size, lack of adequate controls, and of conclusive screening for secondary hypertension.

**Figure 2.**
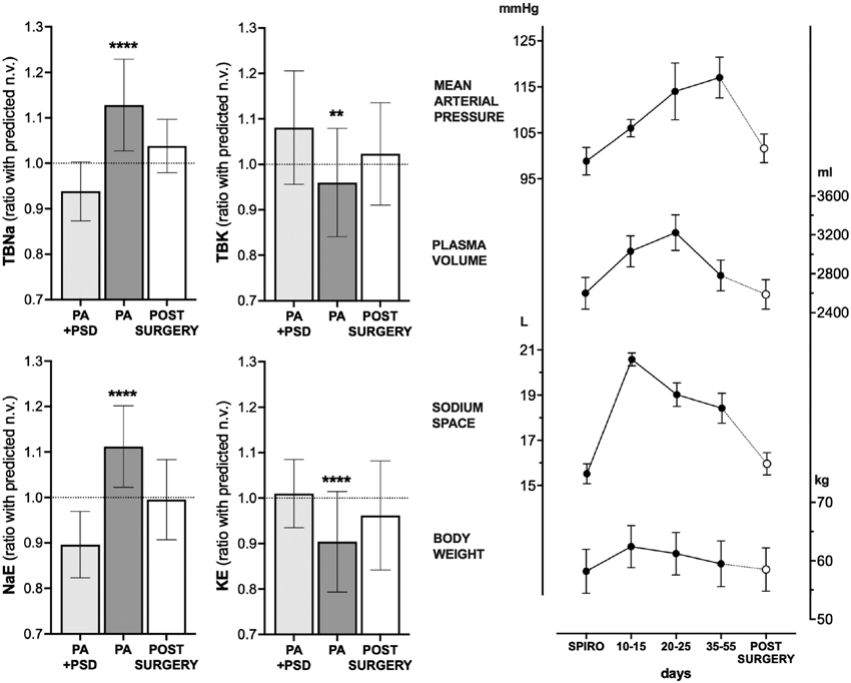
**Sodium, potassium, and fluid balance in primary aldosteronism. Left**, Nuclear whole-body counting, revealing high total body Na^+^ (TBNa) and exchangeable Na (NaE) in patients with an aldosterone-producing adenoma without treatment (primary aldosteronism [PA]); both were reduced by spironolactone or amiloride (potassium-sparing diuretics; PSD) and by adrenalectomy. Total body K^+^ (TBK) and exchangeable K^+^ (KE) revealed opposite trends. Data are presented as mean±SD. **Right**, Changes induced in 5 patients with PA by withdrawal of spironolactone and by adrenalectomy. **P<0.01, ****P<0.0001 vs normal values. Drawn from Williams et al.^78^ Copyright © 1984, Lippincott-Raven Publishers. Data are presented as mean±SEM. Adapted from Wenting et al^79^ with permission. Copyright ©1977, Wolters Kluwer Health.

PA, a prototypic form of salt-dependent hypertension, has offered valuable insights into these aspects over the years. Conn studied isotope dilution over 28 days in 6 patients with PA and reported evidence of a diminished NaE pool that did not readily equilibrate within 24 hours.^75^ This was interpreted as diminished bone sodium, in keeping with the later recognition of a chronic bone-resorptive state, driven by the hypercalciuria and hyperparathyroidism, typical of PA.^76,77^ Such a diminished slowly exchangeable pool could not be confirmed by later approaches of noninvasive whole-body counting,^78^ but these were hampered by a one-off, relative rather than absolute, overall complex and likely less sensitive nature of the methodology, compared with a 4-week daily blood dilution measurement. Nevertheless, they sufficed to provide compelling evidence of high total body Na^+^ and NaE in PA, which were reduced by spironolactone or amiloride and by curative surgery (Figure 2, left).^78^ Almost 30 years before seeing tissue Na^+37^ we knew it was there, somewhere.

## SHRINKING BUCKETS

Recent work, only apparently unrelated with aspects pertaining Na^+^ localization, linked excess sodium to a metabolic shift toward catabolism. The authors of the original observation noted a decrease in water intake, without changes in urine volume, during high compared with low salt intake in the aforementioned human long-term balance studies.^80^ Parallel experiments in rodents exposed to (admittedly extreme) salt loading, including pair-feeding approaches, revealed surplus generation of endogenous water by muscle protein catabolism and hepatic ureagenesis, coupled with reduced free water clearance by urea-driven water reabsorption in the kidneys.^81^ Analogy was made with animals experiencing dormancy during conditions of aridity and high temperatures, suggesting a multiorgan water conservation system to prevent natriuresis-induced water loss during high Na^+^ intake. More recently, the same authors reported that these mechanisms operate also in other experimental models prone to dehydration, like impaired urine concentration ability in 5/6 nephrectomy,^82^ vasopressin antagonism,^83^ and psoriatic skin barrier defect.^84^ Others independently confirmed that mild water deficit achieved by chronic restriction shifted metabolism toward catabolic water production, increased energy expenditure, and high food intake, ultimately shortening life span in mice.^85^ In a retrospective analysis of patients with essential hypertension, we identified similar water-preserving mechanisms upon high Na^+^ intake, arguably sustained by energy-consuming renal changes and characterized by peripheral metabolomic signatures suggestive of protein catabolism.^86^ Similarly, a high-salt diet decreased free water clearance and increased the excretion of amino acids involved in the urea cycle in a randomized trial including 20 lean and 20 abdominally obese individuals.^87^ In fact, a secondary analysis of the DASH (Dietary Approaches to Stop Hypertension)-sodium randomized trial could not confirm different energy requirements across different dietary sodium levels, but weight did vary despite the attempt for controlled energy intake and measures for body composition were missing.^88^

Collectively, evidence suggests that under conditions of salt excess, the cellular mass may shrink because of a water-preserving catabolic state. In free-living conditions, different energetic sources, either endogenous (muscle mass) or exogenous (excess food), can be exploited, thus complicating absolute and relative assessments. Moreover, water balance depends not only on intake, diuresis, and catabolism but also on water in ingested food and exchange via other routes, including respiration, feces, and skin,^84,89^ all substantively affected by environmental and lifestyle factors.^90^

All these considerations are key for interpreting any Tangram compartment puzzle: no surprise that watery volumes or weights were missing, if one accounts for the parallel (subclinical) loss of cellular mass with salt loading. This contention still lacks conclusive experimental confirmation; however, in the representative model offered by PA, simultaneous assessment of the whole-body elemental composition revealed a significant potassium deficit (Figure 2, left), and total body potassium is an old but still one of the most precise measures of cell mass.^1,91^

## TO BE OR NOT TO BE

Changes in the relative proportion of extracellular and intracellular volumes can profoundly impact on whole-tissue Na^+^ content and concentration.^61^ However, uncertainties about cell mass (and intracellular volume, accordingly) are not the sole factor affecting the syllogism that long-term divergence of Na^+^ and water balance would equal hypertonic accumulation.

Our chemical analysis of multiple tissues in salt-loaded rats and of skin biopsies from patients with hypertension, by us and others,^62,92^ did not support the hypothesis of a hypertonic Na^+^ excess, since tissue water largely paralleled tissue Na^+^. We interpreted the findings as a systemic expansion of the extracellular volume, that is, subtle isotonic edema. Interestingly, the isolation of interstitial fluid and lymph draining the skin of rats during salt accumulation induced by a high-salt diet or deoxycorticosterone pellet implantation revealed Na^+^ concentration that was not different from plasma.^60^ While this cannot exclude the preferential binding of Na^+^ to the glycosaminoglycan network in the ECM (extracellular matrix), its quantitative relevance has been challenged,^31^ as discussed above. Moreover, the stoichiometry of any Na^+^ binding in excess of water, which is similarly attracted by glycosaminoglycans, remains unclear. In fact, in agreement with previous data,^60,62,93^ high-salt diet caused an increase in skin water content; the sum of cations remained within physiological ranges in the whole tissue and only modestly increased in the dermis, in parallel with increased Na^+^ concentration in the serum.^31^ This increase is well below those reported in both high salt and control animals in the original studies that suggested Na^+^ hypertonicity.^18^ Moreover, a chemical analysis coupled with extracellular volume tracking with ^51^Cr-EDTA confirmed that the extracellular space undergoes expansion with salt loading, particularly in the loose dermis.^31^ Even in dermis, extracellular Na^+^ concentration never exceeded ≈120 mmol/L, when intracellular Na^+^ was conveniently assumed to be fixed at 10 mmol/L (which may have magnified the extracellular estimates if true intracellular values were higher; see below).

Does this evidence dismantle the concept of hypertonic tissue Na^+^ accumulation? No, it does not. First, except for the specialized renal medulla, there is currently no firm evidence to confirm or exclude that local gradients of Na^+^ exist in tissues. If present, they would likely to be smaller than initially suggested, but lack of magnitude does not equal lack of biological relevance: excess Na^+^ can modulate multiple immune cells and polarize them toward an inflammatory phenotype, as extensively reviewed elsewhere.^94,95^ Furthermore, even mild increases reflecting repeated (dietary) insults^96^ or long-term mishandling may eventually be pathogenic over a life span. Recently, serum Na^+^ values as low as the upper limits of normal were associated with long-term risk of developing left ventricular hypertrophy and heart failure in a population-based prospective cohort study,^97^ consistent with old cellular studies.^98^ Second, the epidermis of salt-loaded rats indeed showed hyperosmolarity but due to osmolytes other than Na^+^, like urea.^60^ This may link with water loss at the epidermal surface^89^ or to counter-mechanisms preserving body water in conditions of water deficit and (relative) Na^+^ excess.^81,84,99^ However, it seems unlikely that this surface hyperosmolarity can substantially affect deeper cells, since the interstitial fluid Na^+^ (and protein) concentration is similar in normal and high-salt conditions.^35,60^ This contention was proven by the evidence of similar, or even reduced, shift of fluids from blood capillaries into the interstitium in human heart failure compared with age- and sex-matched controls.^100^

One last key, previously suggested^1^ but generally neglected aspect to consider is the shift of Na^+^ inside the cells. Recent experimental evidence revealed that skeletal and cardiac muscle of salt-loaded rats indeed accumulate Na^+^, without changes in total Na^+^+K^+^ concentration^31,62^ but with marked increases in intracellular Na^+^ concentration.^31^ Also skin, where ≈25% of the fluid is intracellular,^31^ is a potential compartment for Na^+^/K^+^ exchange and skin cells may act as Na^+^ reservoir that may appear as 'bound' irrespective of sulfated glycosaminoglycans. In fact, the concept is not new in the field of hypertension and there are old reports of excess intracellular Na^+^ in smooth muscle, as well as circulating cells.^101^ Unfortunately, definite conclusions were limited by methodological inconsistencies, which possibly still impact the herein discussed field. We are now aware of multiple molecular mechanisms and pathogenic correlates of excess sodium entering immune cells^95,96^ to support those early descriptive findings. In addition, recent evidence extends the phenomenon to body cell mass at large, including skeletal and cardiac muscle cells, with obvious electromechanical and energetic implications for their function. This makes our Tangram even more complicated.

## FROM A UNIQUE HUMAN MODEL TOWARD A UNIFYING VIEW

PA does not generally present with overt signs of fluid overload, that is, detectable edema. This is explained by the so-called aldosterone escape, whereby administration of large doses of aldosterone does cause an initial decrease in urinary sodium excretion, but this phase is followed by a gradual increase to eventually match intake, thus attaining a new equilibrium and avoiding overt Na^+^ and water retention.^102–104^ This process depends on increased renal perfusion pressure, high sodium delivery to the distal nephron that overrides the usual mineralocorticoid-driven reabsorption, and increased natriuretic peptides.^105^ At least 2 of these mechanisms are driven by volume expansion that a quick clinical look may miss but that original investigators spotted as slight periorbital puffiness.^102^ In fact, in our experience of systematically searching for and subtyping PA,^12^ patients with hypertension who temporarily undergo washout from drugs like mineralocorticoid receptor antagonists do often report some degree of subjective swelling. A small study, conducted on 5 patients with overt PA submitted to a protocol of spironolactone washout in a metabolic ward, revealed that sodium space and exchangeable sodium rose until 10 to 15 days and declined afterward, although eventually remaining higher than during spironolactone treatment (Figure 2, right). Plasma, blood volumes, and body weight returned to values that are only minimally, but not significantly, higher than baseline. All parameters normalized at long-term follow-up after surgery.^80^ Unfortunately, the study did not track K^+^, which (1) is missing in patients with PA at whole-body counting (Figure 2, left)^78^; (2) when expressed as either plasma concentration, exchangeable K^+^, or total body K^+^, correlated inversely and significantly with BP in patients with hypertension^71^; (3) at variance with Na^+^, never reached a new equilibrium between excess excretion and intake in the early escape experiments.^103^ Although it would be key to know whether the cell mass shrank in those 5 patients on a fixed diet over the course of the almost-2-month washout, evidence for a parallel weight loss in PA remains anecdotal,^103^ conflicting for plasma volume,^106–108^ but certainly conclusive for an expanded NaE, Cl^−^, and extracellular fluid volume.^106,107,109,110^ The phenomenon may not be restricted to a prior history of hypertension^80^ and recapitulate the cardiovascular continuum that spans from a variety of subclinical states to overt interstitial congestion, or heart failure, to which PA demonstrates high risk of progression.^111^ Similar to PA, all these pre-heart failure states (eg, resistant hypertension, diabetes, CKD) feature excess tissue Na^+^ at ^23^NaMRI. Additionally, in the context of metabolic syndrome or CKD, absolute or relative cell mass reduction is both a determinant and a result of disease.^112,113^ Whether this is also paralleled by intracellular Na^+^ accumulation remains to be established, but the high ^23^Na signal from skeletal muscle,^38,41,53,55^ in addition to the dermis, would suggest so.

**Figure 3.**
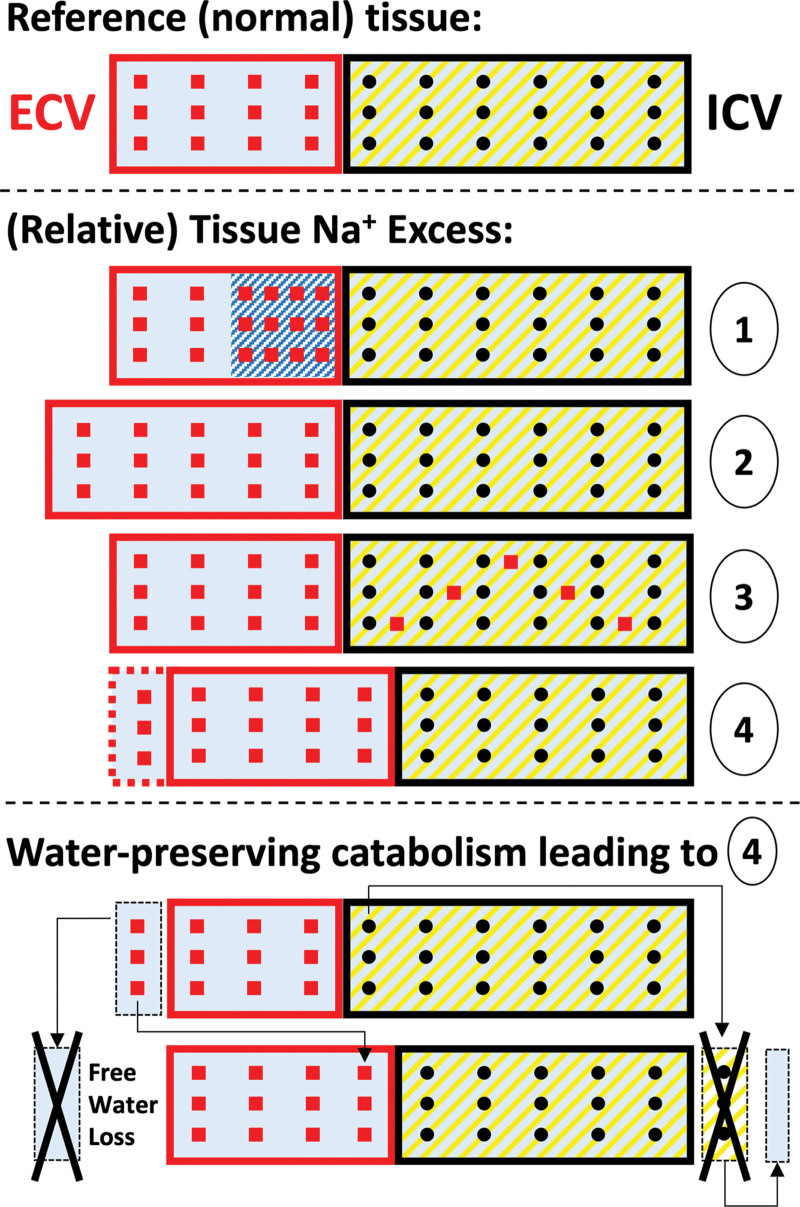
**Reappraisal of the Tangram for interpretation of tissue Na excess. Top**, Physiological reference tissue, composed of extracellular and intracellular volumes (ECV and ICV), rich in Na^+^ (squares) and K^+^ (dots), respectively. **Middle**, Different pathophysiological patterns resulting, at whole-tissue analysis (eg, in ^23^NaMRI), in tissue Na^+^ excess: (1) hypertonic tissue Na^+^ accumulation, whereby Na^+^ would bind glycosaminoglycans (shaded) in the extracellular matrix in excess of water; (2) absolute expansion of the ECV, that is, edema; (3) accumulation of Na^+^ inside the cells, as for muscle; (4) relative but not absolute ECV predominance due to shrinking ICV (with or without additional edema). These patterns may coexist in different disease states. **Bottom**, Suggested mechanism described in long-term/experimental excess Na^+^ intake^80,81^ and other water-losing conditions,^82–84^ by which free water deficit or loss would induce a catabolic state and a loss of cellular mass and of K^+^, resulting in relative tissue Na^+^ excess, in the attempt to generate endogenous water moieties from the breakdown of proteins. Loss is depicted as a black X.

In summary, our interpretation of the tissue Na^+^ excess deserves a reappraisal of the Tangram: (1) small interstitial hypertonic niches, (2) more or less clinically visible edema, (3) intracellular Na^+^ accumulation, (4) or relative cell mass loss (driven by aging, whole-body sodium-water imbalance, and the ensuing water-preserving catabolic state) may all present with the final readout of excess tissue Na^+^ signal (Figure 3).

## OPEN MIC! MICROVASCULATURE, INTERSTITIUM, AND CELLS

We propose a small functional unit to interpret excess tissue Na^+^ in hypertension and cardiovascular disease. This MIC unit includes (1) the blood and lymphatic microvasculature (M) down to capillaries, which are responsible for fluid extravasation and removal, respectively; (2) the interstitium (I), a highly dynamic interface; (3) parenchymal cells (C), which are impacted by but also active determinants of the extracellular—and ultimately whole body—Na^+^ content (Figure S4). Each component of the MIC unit has either been discussed above (shrinking buckets) or reviewed elsewhere in relation to Na^+^ handling by the blood vessel wall and its permeability,^114^ the role of lymphatics in interstitial homeostasis, the composition and biophysics of the interstitial matrix, and the local forces that impact stromal and immune cells.^1,115–118^

Despite the unique sodium handling by salt-sensitive and salt-resistant subjects,^119–121^ all these players have received little experimental attention due to a difficult investigation and the wrong belief that there was little left to discover.^1^ This hasty conclusion disregards historical evidence. Tarazi et al^122,123^ found that the ratio of plasma volume (PV) to interstitial fluid (IF) volume was significantly lower in uncomplicated, untreated essential hypertensive patients compared with normotensive subjects. The difference was independent of diminished PV, rather indicating a shift of extracellular fluid from the intravascular to the interstitial compartments. In patients with variable degrees of CKD, salt loading consistently expanded the extracellular volume; however, the PV/IF ratio decreased in those with mild estimated Glomerular Filtration Rate (eGFR) reduction and increased in those with more severe impairment. PV/IF change directly correlated with the change in BP (Figure S5).^124^ In rats, undergoing sequential removal of both kidneys, Lucas and Floyer^125^ found changes in the PV/IF ratio similar to those in patients with more severe CKD but also a marked increase in interstitial tissue pressure and a fall in interstitial compliance after saline infusion. A similar pattern was found in one-kidney one-clip rats,^126^ suggesting modulation of those parameters and their determinants by the renin-angiotensin-aldosterone system. Later studies (in which both PV and IF volume were directly measured, rather than calculated from changes in total extracellular volume) confirmed different changes in PV during experimental dehydration and fluid load but not different interstitial compliance in one-kidney one-clip rats compared with one-kidney sham-clipped normotensive controls.^127^ However, the interstitial pressure and volume at baseline were higher in hypertensive compared with control rats and their change after peritoneal dialysis or the change in the interstitial colloid osmotic pressure after saline load differed. Unfortunately, there was no follow-up to these old studies. All in all, available data suggest different tissue-capillary filtration forces and dynamics in different patients of the cardiovascular-renal spectrum of hypertensive disease.

## SUMMARY AND CLINICAL PERSPECTIVES

There is no polished surface (Supplemental Material S6) in the field of sodium balance. The old intracellular-extracellular 2-compartment model of fluid and electrolyte homeostasis was first expanded to 3 compartments, in which the interstitium featured as a separately regulated space. Our body’s Na^+^ balance was found to disregard the need for strict equilibrium not only on a day-to-day basis but also in the ultra-long term: Na^+^ accumulates in tissues with aging, cardiorenovascular and inflammatory diseases. Investigators from the 70s could already guess it, but recent technological advancement, namely ^23^NaMRI, enabled us to see the invisible. At odds with its initial intended use for the extracellular space, the anatomic approach of ^23^NaMRI led us to further rethink the 3-compartment model and dignify the intracellular space. For too long regarded as a constant in the equation, intracellular volume emerged as (1) a key determinant of the architecture of tissues and of their total chemical composition; (2) the immediate target of catabolic processes triggered by conditions of water and Na^+^ imbalance; (3) one additional key site for tissue Na^+^ accumulation, particularly in muscles but also skin, with electromechanical implications that remain to be unraveled. Finally, we realized that at least part of the interstitial Na^+^ storage is not independent of water, in keeping with old beliefs.

Even reshuffling this Tangram, thanks to a decade of progress built on the shoulders of giants, still does not fill many of the blanks. We can now see and better interpret tissue Na^+^ excess based on evidence-based pathophysiological patterns (Figure 3); however, we do not know which (or which combination) best applies to each timing of each disease. We have proposed the MIC unit to help researchers, and hopefully clinicians in the future, to disentangle the crucial interplay of physics, biomechanics, and energetic balance at such a microscopic scale and understand their full therapeutic implications.

One last, but compelling and overarching question remains: does sodium and water retention, even at subclinical scale, affect organ function, translate into organ damage, and ultimately affect prognosis? Data from patients with PA, herein referenced as the epitome for the discussed aspects, would suggest so. Although traditionally considered a benign form of hypertension because of the undetectable renin levels,^128^ extensive work from us and others has robustly associated PA with excess left ventricular hypertrophy, LV fibrosis, vascular remodeling, microalbuminuria, endothelial dysfunction, and with a high risk of stroke, myocardial infarction, heart failure, and atrial fibrillation.^112^ Of note, surgical cure of PA was associated with a decrease of incident atrial fibrillation and regression of left ventricular hypertrophy via reverse inward remodeling,^129^ in line with the concept that removal of the mineralocorticoid-mediated salt-retaining excess is associated with a decrease of body fluid volumes. Such evidence from this salt-dependent and reversible disease model may guide the investigation of the molecular mechanisms implicated in the pandemic of tissue Na^+^ excess. In perspective, it may be relevant to more precise treatment of many other cardiorenovascular diseases and possibly beyond.

## ARTICLE INFORMATION

### Sources of Funding

G. Rossitto receive support from the NextGenerationEU-funded program STARS@UNIPD 2021 (starting grant; POLYPHEMUS-CVD); G. Rossitto and G.P. Rossi from the University of Padua, and the Foundation for Advanced Research in Hypertension and Cardiovascular Diseases; C. Delles from the British Heart Foundation (Center of Research Excellence; RE/18/6/34217); H. Wiig from the Research Council of Norway (project number 262079), the Norwegian Health Association, and from the Western Norway Regional Health Authority (project number 912168).

### Disclosures

None.

## Supplementary Material


