# Supplementary material for: Tangram of Sodium and Fluid Balance
Source: Hypertension. 2023 Dec 12;81(3):490–500. doi: 10.1161/HYPERTENSIONAHA.123.19569 (PMC10863667; doi:10.1161/HYPERTENSIONAHA.123.19569)
Supplement: Supplementary file 1 [file hyp-81-490-s001.doc]

**The Tangram of Sodium and Fluid Balance**

Domenico Bagordo^1^, Gian Paolo Rossi^1^, Christian Delles, Helge Wiig^3^ and Giacomo Rossitto^1,2^

^1^ Emergency and Hypertension Unit, DIMED; Università degli Studi di Padova, Italy

^2^ School of Cardiovascular & Metabolic Health, University of Glasgow, UK

^3^ Department of Biomedicine, University of Bergen, Norway

**SUPPLEMENTAL MATERIAL**

Corresponding author:

Dr Giacomo Rossitto

Emergency Medicine and Hypertension,

Department of Medicine, Università degli Studi di Padova,

Ospedale Universitario, Via Giustiniani 2,

35128 Padova, Italy

e-mail: [giacomo.rossitto@unipd.it](mailto:giacomo.rossitto@unipd.it)

**Figure S1.**

**Variability of sodium excretion around fixed intake.** Daily urine collection for Na^+^ excretion (UNaV; red) and recorded sodium intake (mmol/d, black) of all subjects involved in the long-term balance studies,^4^ who consumed menus with fixed Na content (12, 9 and 6 g salt per day; dashed white lines); systematic average difference = -12±39 mmol/d. Adapted from *Lerchl K et al, Hypertension. 2015* ^3^.

**Figure S2.**

**Nomogram for conversion of Sodium (Na^+^) and Salt (NaCl) amounts.**

Recommended intake thresholds are indicated.

**Figure S3.**

**
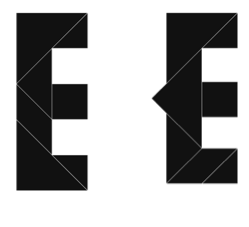
**

**Solution to the apparent paradox in Figure 2.** A tangram paradox is a dissection fallacy: two figures composed with the same set of pieces, one of which seems to be a proper subset of the other.

The paradox remains incomprehensible only if one doesn’t consider that the constituting pieces can “move” and that tiny but significant volume differences may elude detection.

**Figure S4.**

**MIC unit.** Na^+^ balance in tissues reflects the interplay between microvascular fluid exchange (M), stromal cells and matrix in the interstitium (I), as well as volume and content of parenchymal cells (C). Fluids and solutes, including Na^+^, are filtered through the arterio-venous (A-V) capillary wall, based on microvascular permeability (K) and the net result of hydraulic and osmotic pressure gradients between lumen and interstitium (ΔP - Δ∏). Accumulation of interstitial Na^+^ and water is favored by their binding to the negatively charged glycosaminoglycans (GAG), for which an active regulation by salt remains contentious, and opposed by the collagen fibers (Coll) anchored to fibroblasts (F). The resulting osmotic, electrochemical and hydrostatic forces impact on the stromal cells, including F and immune cells (IC), and parenchymal cells. Interstitial mechanics also explain the entry of fluids and solutes into lymphatics capillaries (J_L_), for their ultimate drainage by the lymphatic system. Parenchymal cells actively contribute to this homeostasis by exchanging water and electrolytes, but also proteins and other osmolytes, with the interstitium: not only Na^+^ increases over K^+^, as a total pool, when the cellular mass is reduced, but it also accumulates in excess in the intracellular space after salt loading, particularly in case of mineralocorticoid excess.

**Figure S5.**

**Relationship between the distribution of fluids in the extracellular space and salt sensitivity.**

Redrawn from *Koomans HA et al, Hypertension 1982; 4:190-197*. Change in plasma volume/interstitial-fluid volume (PV/IF) ratio and in salt sensitivity index after increased salt intake, in patients with different degrees of renal impairment (**blue**: patients with mild renal impairment, Creatinine clearance = 47.0 ± 15.7 ml/min; **black**: patients with severe renal impairment, Creatinine clearance = 9.5 ± 5.5 ml/min). The blood pressure (BP) salt sensitivity index, which increased exponentially with decreasing creatinine clearance, was calculated from the increase in mean arterial pressure accompanying a certain amount of sodium excretion increase. ρ = 0.60, p < 0.01.

**Supplemental quote**

“*After conducting his research amid discoveries and bewilderment (perhaps filling in blanks present at the outset, at times leaping rightly from one idea to another, checking hypotheses and then discarding them) a researcher really need not finally close by offering the reader a most* ***polished surface****, a straightforward and coherent argument, where everything seems almost miraculously to fall into place and the author seems to speak with the Olimpian authority of an all-seeing all-knowing god*”.

Foreword by Umberto Eco. In: *On the shoulders of giants: a Shandean postscript, Post-*

*Italianate ed., University of Chicago Press ed. Chicago: University of Chicago Press; 1993*.
